# Plasma levels of phosphorylated tau and neurofilament light chain as potential biomarkers for Alzheimer’s disease: A biochemical analysis in Pakistani population

**DOI:** 10.1016/j.jsps.2023.05.013

**Published:** 2023-05-18

**Authors:** Tehniat Faraz Ahmed, Muhammad Bilal Azmi, Fauzia Imtiaz, Uzma Zaman, Affan Ahmed, Naila Shahbaz

**Affiliations:** a Department of Biochemistry, Dow International Dental College, Dow University of Health Sciences (DUHS), 74200 Karachi, Pakistan; b Department of Biochemistry, Dow Medical College, Dow University of Health Sciences (DUHS), 74200 Karachi, Pakistan; c Department of Biochemistry, Dow International Medical College, Dow University of Health Sciences (DUHS), 74200 Karachi, Pakistan; d Dow Medical College, Dow University of Health Sciences (DUHS), 74200 Karachi, Pakistan; e Department of Neurology, Dr Ruth Pfau Civil Hospital Karachi, 74400 Karachi, Pakistan

**Keywords:** Alzheimer disease, Biomarkers, Plasma, Amyloid beta-peptides, Tau proteins, Neurofilament protein L, Enzyme-linked immunosorbent assay

## Abstract

The National Institute on Aging-Alzheimer's Association's research framework in 2018 proposed a molecular construct for the diagnosis of Alzheimer’s disease (AD). Nonetheless, the clinical exclusionary strategy is still the mainstay of AD diagnosis in Pakistan. We looked at the plasma levels of amyloid beta-42 (Aβ-42), phosphorylated tau (P-tau), and neurofilament light (NFL) in patients with Alzheimer's clinical syndrome (ACS) and healthy controls (HC) from the Pakistani population to keep pace with the global efforts towards establishing accessible and affordable biochemical diagnostic markers for AD in Pakistan.

Consultant neurologists screened patients who presented with cognitive impairment to three large tertiary care hospitals in Karachi, and after receiving informed consent, recruited participants with ACS and HC from the same facilities. We collected 5cc of blood in EDTA tubes along with demographic and lifestyle information of the subjects. Plasma aliquots were stored at −80°C after centrifugation. For analysis it was thawed at 4℃ and levels of the three proteins were measured through ELISA.

Data from 28 ACS patients and 28 age matched healthy controls were evaluated. Among demographic factors, *education* and *depression* were related with health status (*p* = 0.03 and 0.003, respectively). NFL and P-tau mean values demonstrated a significant difference between the ACS and control groups (*p* = 0.003 and 0.006), however Aβ42 did not (*p* = 0.114). ROC analysis showed that plasma P-tau and NFL, with AUCs of 0.717 and 0.735, respectively, could substantially distinguish ACS from the HC group (*p* = 0.007 and 0.003, respectively). Both plasma P-tau (*r* = −0.389; *p* = 0.004) and NFL (*r* = −0.424; *p* = 0.001) levels were significantly and negatively correlated with individuals' MMSE scores. NFL and plasma P-tau show promise in differentiating AD patients from healthy individuals. However, similar larger studies are needed to validate our findings.

## 1 Introduction

Alzheimer’s disease (AD) is the most common form of dementia, which is characterized by deficits in processing and storing new information, followed by cognitive and behavioral changes later in the disease course (Soria Lopez et al., 2019). With increasing life expectancy, the threat of AD is growing at an alarming rate. As indicated in the World Alzheimer Report 2019, 46.8 million people are currently living with AD, and this number will triple by 2050 as the population ages (Lynch 2020). No epidemiological studies have been conducted in Pakistan to measure the burden of AD; however, the 2019 Global Burden of Disease study estimates 323,669 people (0.15%) living with Alzheimer’s disease and other neurodegenerative dementias in Pakistan (Global Health Data Exchange 2019).

Alzheimer's disease is characterized by abnormal cleavage of Amyloid Precursor Protein (APP) by beta-secretase, which results in the extracellular accumulation of insoluble amyloid beta plaques. This and other factors drive intracellular neurofibrillary tangles (NFT) formation comprised of hyper-phosphorylated and truncated tau proteins (Gallardo and Holtzman 2019). Phosphorylated-tau (P-tau) has prion-like activity and polymerizes to form paired helical filaments that aggregate to produce neurofibrillary tangles (Alonso et al., 2018). These pathological processes in the brain cause neuronal degeneration and synaptic loss, culminating in macroscopic atrophy of the affected brain regions (Lane et al., 2018).

Mixed etiology, overlapping symptoms, variation in presentation, and a lack of standardized, accessible biomarkers render AD still difficult to diagnose (Ahmed et al., 2021). Even after more than a century of rigorous research, no disease-modifying treatment yet exists, and only symptomatic management is provided to AD patients (Golde 2022). This high failure rate of clinical trials can be attributed to pharmacological intervention late in the disease course. Since it is strongly suggested that treatment is rendered ineffective after a certain pathological threshold, there is a pressing need for early diagnostic markers of AD (Mossello and Ballini 2012). The research framework articulated by the National Institute on Aging–Alzheimer’s Association (NIA-AA) envisions AD as a molecular rather than a clinical construct. This scheme proposes the use of PET scanning and CSF analysis for biochemical identification of AD and divides the biomarkers into amyloidosis (A), tauopathy (T), and neurodegeneration (N) categories (Jack et al., 2018). However, the projected benefits of biomarkers have not been achieved so far owing to their invasiveness and high cost. Blood-based biomarkers represent a significant substitute in a primary care setting (Zetterberg and Burnham 2019).

AD has been identified as a global public health priority, with the greatest impact expected in low- and middle-income countries (11). We conducted the first study of its kind to investigate non-invasive screening tests for the diagnosis of Alzheimer's Disease in Pakistan. This is particularly important because there has been a lack of previous biomarker studies for AD in the country, which has limited the ability of healthcare professionals to accurately diagnose the disease. Using the NIA-AA research framework as a guide, we chose amyloid beta 42 from the A category of the AT(N) classification and phosphorylated tau from the T category for in vivo analysis in AD patients and healthy controls. Markers of neurodegeneration are not specific for AD but are indicative of disease severity. We included NFL in our analysis as its use as a biomarker candidate has been supported by several studies and is reflective of neurodegeneration (Lewczuk et al., 2018, Ashton et al., 2021).

## 2 Materials and methods

### 2.1 Selection and stratification of study participants

Adults more than 50 years of age presenting with cognitive decline to the outpatient departments of three public sector tertiary care hospitals in Karachi, were assessed by consultant neurophysicians between May and October 2021. All patients were subjected to Mini-mental state examination (MMSE), which is a simple tool and has a validated Urdu version, to assess cognitive status of individuals. An MRI was conducted if the procedure was not completed within two months to assess brain structure and rule out structural and vascular causes of dementia. Baseline investigations, along with vitamin B12 and thyroid levels were checked to exclude other causes of dementia. Patients with a history of sudden onset of cognitive decline, severe brain injury, psychiatric disease, TIA/CVA followed within 3 months by cognitive decline, normal pressure hydrocephalus, brain tumor, vitamin B12 deficiency, or thyroid disorders were excluded from the study.

**Fig. 1:**
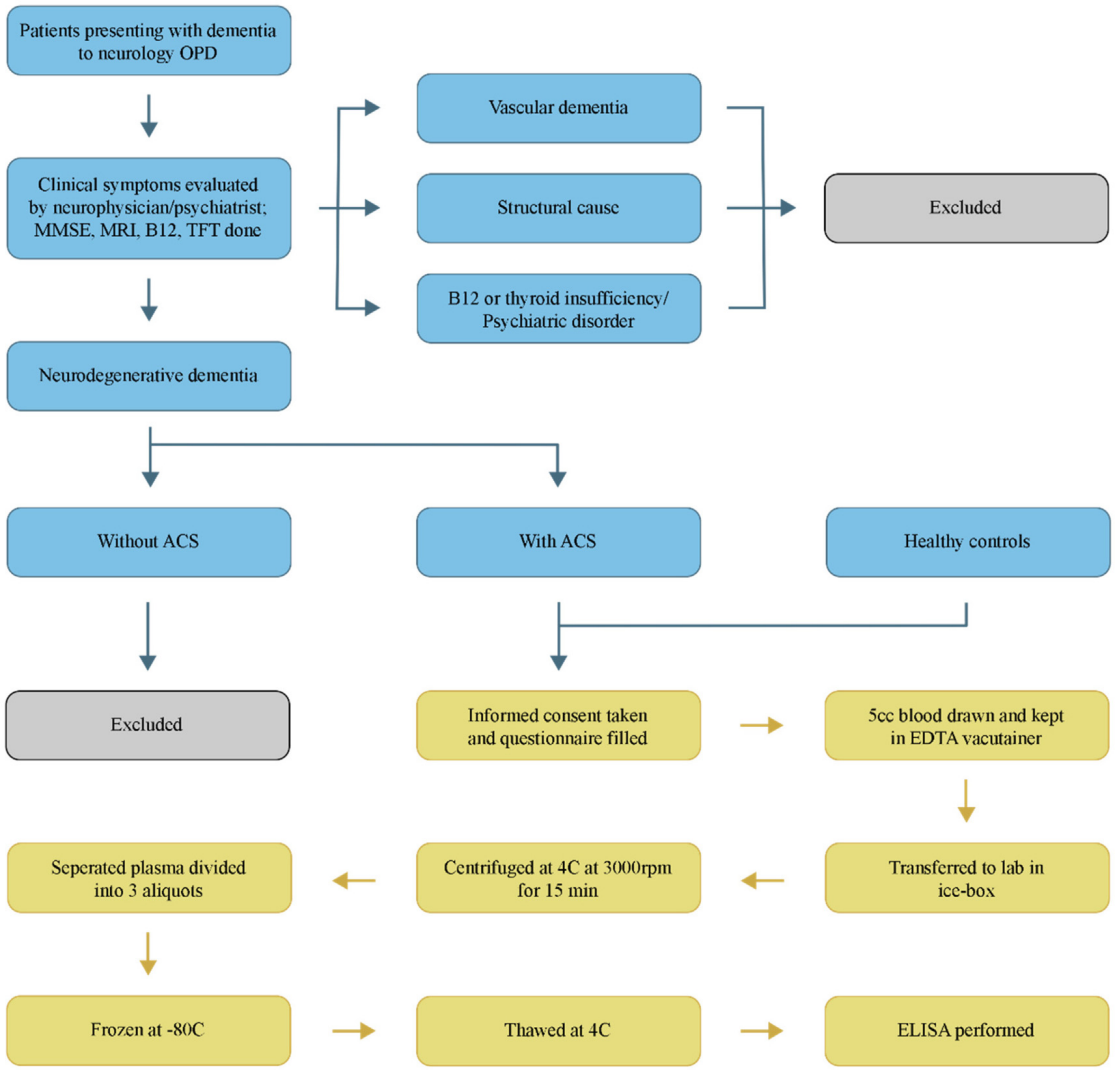
Summary of the methodology adopted during the study: inclusion and exclusion of cases, sample collection, storage, and analysis procedure. ACS: Alzheimer’s Clinical Syndrome.

Those diagnosed with probable AD in accordance with the AD diagnostic criteria published by the NIA-AA in 2011 and the DSM V guidelines (American Psychiatric Association, 2013) were referred to as having Alzheimer’s Clinical Syndrome (ACS) as suggested in the NIA-AA 2018 research framework (McKhann et al., 2011, Jack et al., 2018). DSM-5 guidelines involved clinical evaluation and cognitive testing and provided a well-established and validated criteria for AD diagnosis. Cognitively healthy controls (HC) were individuals with normal cognitive status and were recruited from the same healthcare facilities as the ACS group. Controls were individually age matched with subjects in ACS group. Methodology of the study has been summarized in Fig. 1.

Although Alzheimer’s disease can now be diagnosed on the basis of CSF biomarkers (Jack et al., 2018), a clinical exclusionary diagnosis was chosen as the reference standard. There are no dementia care centers in Pakistan, and Alzheimer’s disease patients could only be recruited from neurology or psychiatry outpatient departments. Lumbar puncture would have resulted in a high refusal rate in patients presenting to OPDs with cognitive decline. This was noted in a pilot survey done by the PI prior to conducting the study. Lack of accessibility to PET scans led to inability to use this method as well. This limitation has been recognized by the NIA-AA in their research framework and encourages the clinical diagnosis of the classic dementia syndrome in settings where stratification based on CSF markers is not possible (Jack et al., 2018).

### 2.2 Ethical considerations

The study was approved by the Institutional Review Board of the Dow University of Health Sciences (Ref: IRB-1999/DUHS/Approval/2021/418). All procedures were in alignment with the code of ethics given by the World Medical Association (Declaration of Helsinki). Prior to their recruitment into the study, all subjects and their attendants were given a thorough explanation of the procedure, and their informed written consent was obtained.

### 2.3 Sample collection

All participants or their attendants were required to fill out a questionnaire to collect their demographic and lifestyle details. Following standard operating procedures of venipuncture, 5cc blood was drawn from each subject and placed into a vacutainer containing EDTA as an anticoagulant. To ensure proper EDTA mixing, the tube was gently inverted five times immediately. All samples were collected between 9 A.M. to 12 noon to exclude the possibility of differences in protein concentration due to diurnal variation. Samples were then centrifuged at 4°C at 3000 rpm for 15 min, and the collected supernatant was aliquoted into 3 polypropylene tubes. Plasma aliquots were stored at −80°C in a cryobox for a range of 24–118 days (median 66 days) pending further analysis through ELISA.

Before analysis, samples were thawed on ice and brought to room temperature. ELISA analysis was performed on a Thermoscientific Spectrophotometer Multiskan™ Sky (Model number 51119700), and the absorbance (ODs) of all experiments was recorded at 450 nm. Plasma Aβ1-42 and Neurofilament Light Polypeptide levels were determined using a human ELISA kit (catalogue number CEA946Hu) and an immunoassay kit (catalogue number SEE038Hu) purchased from USCN Business Co. Ltd., respectively. Plasma P-tau levels were obtained using a human phosphorylated microtubule-associated protein tau ELISA kit (catalogue number E4844Hu) purchased from Bioassay Technology Laboratory. All procedures were carried out according to the manufacturer’s protocol.

### 2.4 Data analysis

The data was analyzed using IBM SPSS version 26.0. Demographic variables were summarized using frequency tables and compared through the Chi-squared test. Protein plasma concentrations of the groups were compared using Mann Whitney *U* test and expressed as mean ± SD. Pearson’s correlation coefficient was computed between the plasma concentrations of the 3 proteins and the MMSE scores of the subjects. ROC analyses were performed to find cut-off concentrations of Aβ42, P-tau, and NFL.

## 3 Results

**Fig. 2:**
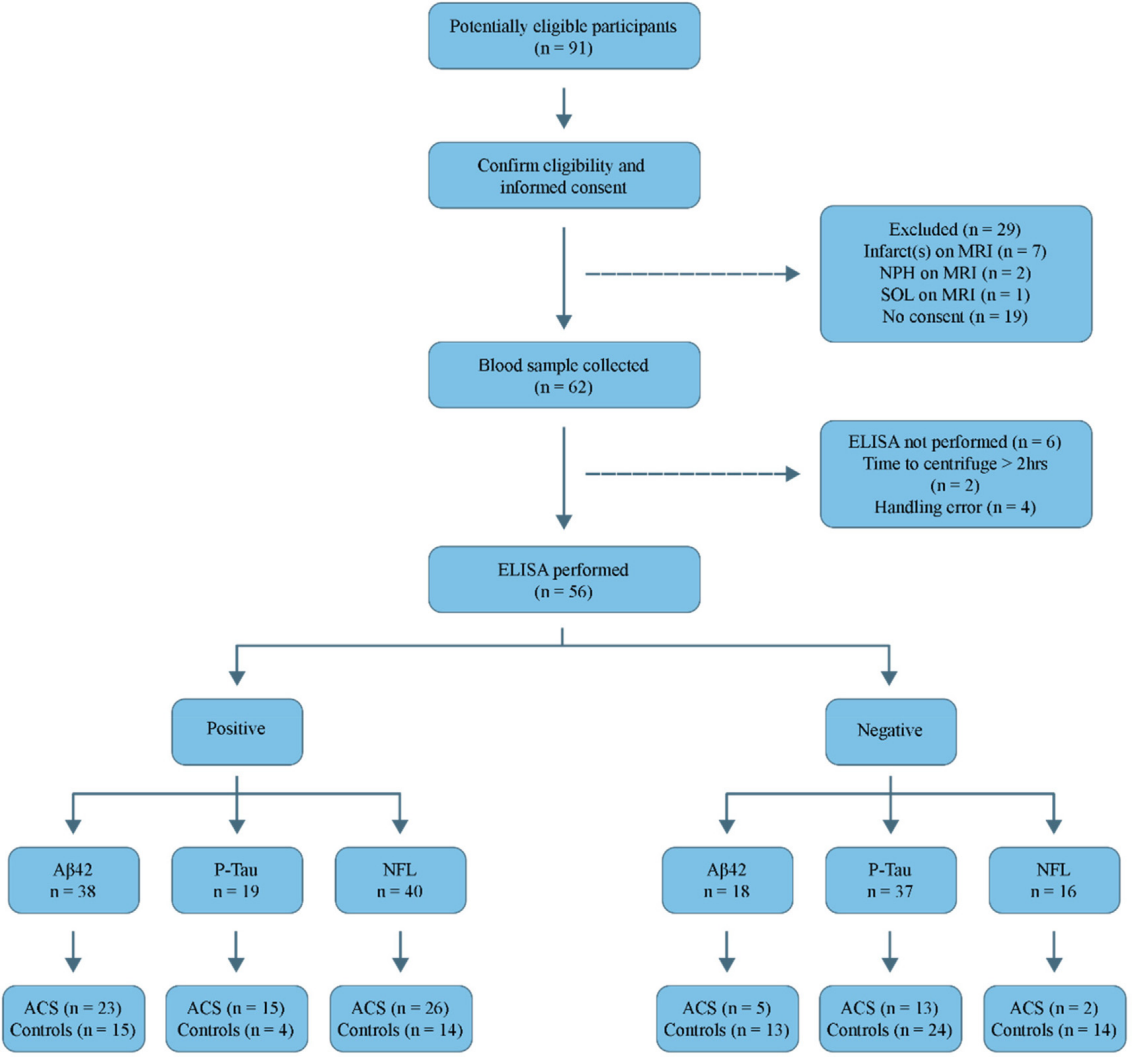
STARD diagram reporting flow of participants through the study.

In total, 56 participants completed the study. Fig. 2 presents the flow of participants as well as the reasons for their exclusion or withdrawal.

The study cohort consisted of 56 age-matched subjects, with 28 in each group of ACS and HC. The demographic features of the study population have been summarized in Table 1. The comparison of the ages of the two groups did not show a significant difference (*p* = 0.516). 68% of the patients within the ACS group were females. Most of the participants in the ACS were Urdu speaking (57%) and had not received any formal education (36%). Depression (61%) and hypertension (50%) were the most prevalent comorbidities in ACS group, with the prevalence of depression being significantly higher in the ACS group as compared to the controls (*p* = 0.001).

**Table 1:** Demographic and health status data of study subjects. Asterisk (*) indicates significant values.

**Parameters**	**N (%)** **(total = 56)**	**CU** **(n = 28)**	**ACS** **(n = 28)**		**P-value**
			MCI	SCI	
**Age (mean years ± SD)**		66.18 ± 10.26	68.11 ± 11.75		0.516
**MMSE (mean score ± SD)**		28.4 ± 1.4	14.8 ± 8.4		
**Gender; n (%)**					0.170
Female	33 (59)	14 (50)	6 (21)	13 (46)	
Male	23 (41)	14 (50)	6 (21)	3 (11)	
**Ethnicity; n (%)**					0.043*
Urdu speaking	23 (41)	7 (25)	6 (21)	10 (36)	
Sindhi	17 (30)	10 (36)	4 (14)	3 (11)	
Panjabi	4 (7)	3 (11)	0 (0)	1 (4)	
Pashtun	7 (12)	4 (14)	2 (7)	1 (4)	
Baloch	5 (9)	4 (14)	0 (0)	1 (4)	
**Education; n (%)**					0.140
No formal education	24 (43)	14 (50)	3(11)	7 (25)	
Primary	13 (23)	8 (29)	2 (7)	3 (11)	
High school	6 (11)	3 (11)	2 (7)	1 (4)	
Graduation	13 (23)	3 (11)	5 (18)	5 (18)	
**Midlife physical activity; n (%)**					0.719
5+ hours	13 (23)	7 (25)	3 (11)	3 (11)	
3–5 h	18 (32)	10 (36)	3(11)	5 (18)	
2–3 h	25 (45)	9 (32)	5 (18)	6 (21)	
1–2 h	7 (13)	2 (7)	1 (4)	2 (7)	
**Diabetes; n (%)**	17 (30)	9 (32)	3 (11)	5 (18)	0.771
**Hypertension; n (%)**	24 (43)	10 (36)	4 (14)	10 (36)	0.280
**Hyperlipidemia; n (%)**	11 (20)	4 (14)	3 (11)	4 (14)	0.313
**Depression; n (%)**	22 (39)	5 (18)	8 (29)	9 (32)	0.001*
**Family h/o dementia; n (%)**	10 (18)	3 (11)	3 (11)	4 (14)	0.163
**Smoking; n (%)**	18 (32)	8 (29)	2 (7)	8 (29)	0.567

The mean plasma Aβ42 concentration was lower in the ACS group (33.33 pg/ml ± 18) as compared to controls (41.38 ± 19.13), but the difference was not statistically significant (*p* = 0.114). The mean plasma concentrations of NFL and P-tau in the ACS group were significantly higher than those in the controls (*p* = 0.003 and 0.006, respectively) (Table 2).

**Table 2:** Mean plasma concentrations of Aβ42, NFL and P-tau in ACS and Control groups.

**Plasma protein**	**ACS group (pg/ml)** **Mean ± SD**	**HC group (pg/ml)** **Mean ± SD**	**P value**
Aβ42	33.33 ± 18.00	41.38 ± 19.13	0.114
NFL	205.10 ± 64.43	145.35 ± 76.27	0.003*
P-tau	265.48 ± 66.68	198.76 ± 96.58	0.006*

When stratified according to their cognitive level, only the plasma NFL concentration was significantly higher in SCI group as compared to those with MCI (*p* = 0.02), while plasma Aβ42 and P-tau concentrations did not differ statistically in patients with mild and severe cognitive impairment.

**Fig. 3.1:**
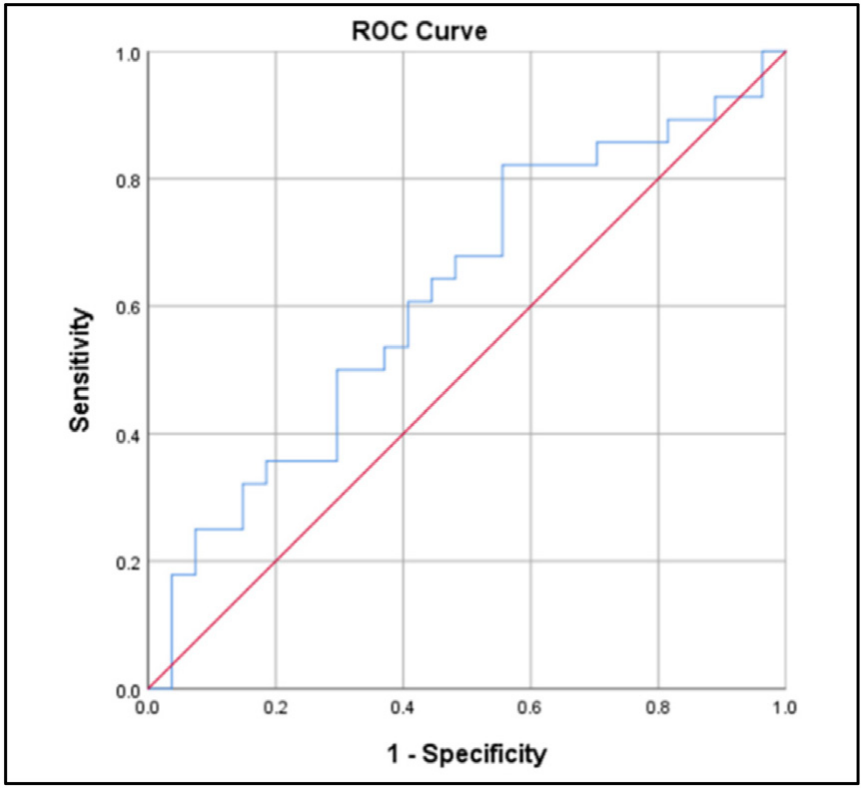
ROC analysis for Aβ42: ACS vs Control group (AUC 0.619; *p* = 0.130).

**Fig. 3.2:**
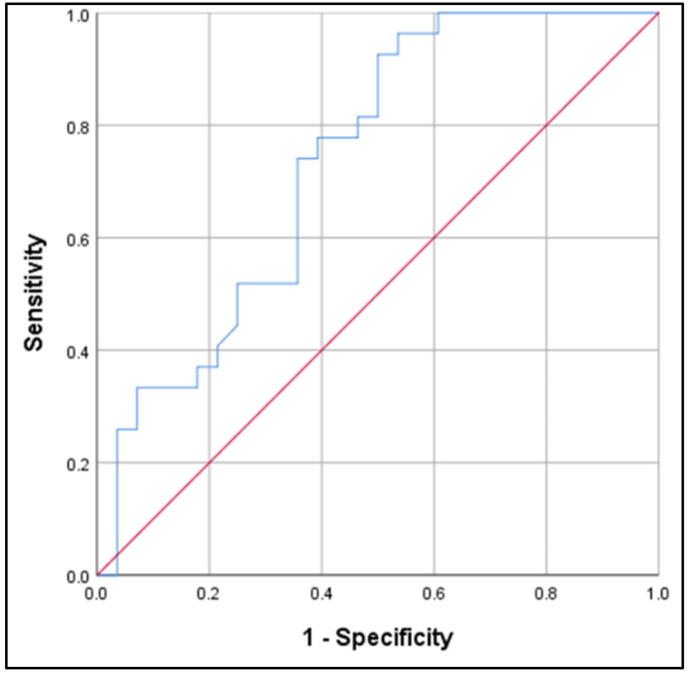
ROC analysis for NFL: ACS vs Control group (AUC 0.735; *p* = 0.003; cut-off 117.47 pg/ml; 93% sensitivity; 50% specificity).

**Fig. 3.3:**
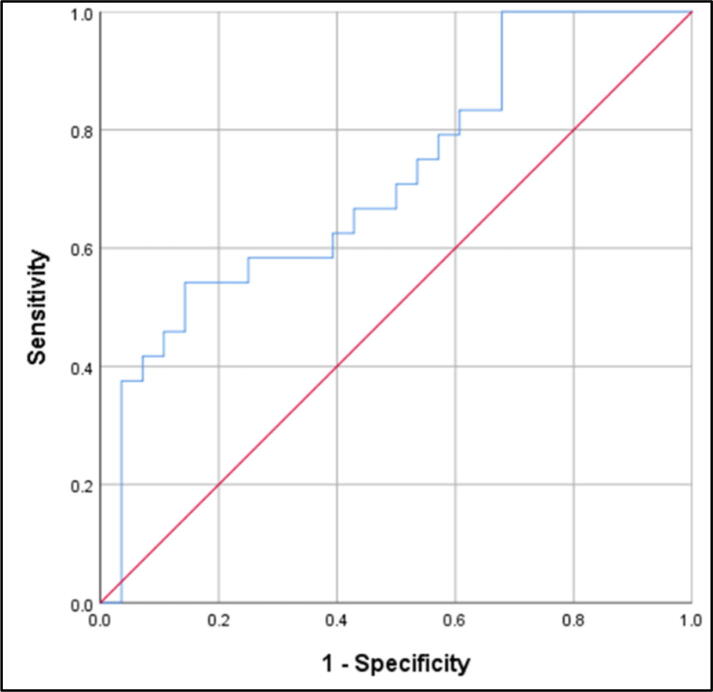
ROC analysis for P-Tau: ACS vs Control group (AUC 0.717; *p* = 0.007; cut-off 271.95 pg/ml; 54.2% sensitivity; 85.7% specificity).

Amyloid β42 did not show promising results for the ACS compared to control group (AUC 0.619; 95%CI 0.470–0.769; *p* = 0.130). Plasma NFL levels above 117.47 pg/ml distinguished ACS from controls with 93% sensitivity and 50% specificity with an AUC 0.735 (Fig. 3.1, Fig. 3.2, Fig. 3.3). Levels of P-tau above 271.95 pg/ml differentiated between ACS and cognitively normal individuals with 54.2% sensitivity, 85.7% specificity, and an AUC 0.717 (95% CI 0.577–0.858; *p* = 0.007). The results suggest the highest diagnostic accuracy of NFL in distinguishing ACS patients from controls.

Plasma NFL levels were found to be inversely correlated with subjects' MMSE scores (*r* = −0.424; *p* = 0.001), as were plasma P-tau levels (*r* = −0.389; *p* = 0.004). The correlation coefficients have been provided in Table 3. No correlation was found between the MMSE scores of subjects and their Aβ42 concentrations (*r* = 0.117; *p* = 0.395).

**Table 3:** Correlation analysis between plasma levels of Aβ42, NFL and P-tau and MMSE score of subjects.

**Plasma protein**	**Correlation coefficient (r)**	**P value**
Aβ42	0.117	0.395
P-tau	−0.389	0.004*
NFL	−0.424	0.001*

## 4 Discussion

According to the World Alzheimer Report 2021, 75% of dementia patients living in developed countries, while up to 90% of those in low- and middle-income countries remain undiagnosed. In the same report, clinicians cited a lack of diagnostic tests as the most significant barrier to dementia diagnosis. Blood biomarkers have recently shown promise in aiding the early diagnosis of dementia (Alzheimer's Disease International 2021). We assessed the plasma levels of three proteins, one from each category of AT(N) biomarkers, in an effort to open the field of biochemical diagnosis of Alzheimer's disease in Pakistan. This was the first plasma biomarker study conducted on this population for the diagnosis of AD.

In this study of 56 participants, 68% of the ACS patients were women. This finding matches the globally reported higher prevalence (7.1%) of AD in elderly females as compared to the prevalence of 3.3% in males of the same age group (Rosende-Roca et al., 2021). The majority of the participants had not received any formal education, which could be explained by the fact that the hospitals participants were recruited from were either in the public sector or had a low fee structure, drawing mostly people of lower socioeconomic status. Frequency of depression was significantly higher in our ACS patients as compared to controls, but longitudinal studies need to be conducted to find the chronological pattern between depression and Alzheimer’s disease.

According to our results, plasma Aβ42 levels did not hold a discriminatory power to assist in the diagnosis of Alzheimer’s disease and had no correlation with the cognitive status of the individual. CSF Aβ42 concentration has been reliably diagnosing AD for quite some time now. However, in the quest for more accessible biomarkers for AD, conflicting results have been reported for plasma Aβ42 concentrations. Several studies have shown decreased plasma Aβ42 concentration and Aβ42/40 ratio in AD patients (Pesaresi et al., 2006, Rembach et al., 2014, Janelidze et al., 2016, Verberk et al., 2018). However, some other studies employing ultrasensitive immunoassays have reported raised Aβ42 levels in AD dementia (Chiu et al., 2013, Lue et al., 2017). Both studies suggested using plasma Aβ42 and tau together for diagnosing AD. Discrepant results can be attributed to several factors, including variation in the age of subjects, dissimilar measurement techniques, and the use of different reference standards for characterizing AD patients. Our results were, however, consistent with the findings of the following studies, which established no correlation between cognitive status of the individual and Aβ levels in plasma and did not they report any difference in the plasma levels of Aβ42 in patients with AD and normal controls (Hansson et al., 2010, Hansson et al., 2012, Donohue et al., 2015). The ELISA technique might not be sensitive enough to detect very low levels of Aβ42 in plasma as compared to highly sensitive techniques such as mass spectrometry. However, the more sensitive techniques are scarcely available and are not suitable for large-scale use in developing countries. More replicative studies are needed to validate the details on the pre-analysis and analysis procedures, which might improve the results obtained through ELISA.

We found significantly raised plasma levels of P-tau in clinically diagnosed AD patients compared to cognitively normal controls. In contrast to Aβ where conflicting evidence is available in terms of Aβ levels, P-tau has been steadily found to be raised in AD patients (Zetterberg et al., 2013, Tatebe et al., 2017, Janelidze et al., 2020, Suárez-Calvet et al., 2020, Thijssen et al., 2020, Hansson et al., 2021). Many studies report similar accuracy of plasma P-tau levels to that of CSF P-tau and tau PET in diagnosing Alzheimer’s disease and differentiating Alzheimer’s dementia from other neurodegenerative dementias (Janelidze et al., 2020, Karikari et al., 2020, Palmqvist et al., 2020, Thijssen et al., 2020).

Due to its consistent results, NFL level in CSF has been recommended for use in clinical practice and clinical research. Several studies have reported similar performance of plasma NFL in differentiating AD from normal controls (Gaiottino et al., 2013, Pereira et al., 2017, Lin et al., 2018, Benedet et al., 2019, Mattsson et al., 2019). According to current data, plasma NFL distinguishes Alzheimer's disease from cognitively normal individuals. NFL is a marker of axonal injury and has been found to be associated with other markers of neurodegeneration (Pereira et al., 2017, Mattsson et al., 2019, Olsson et al., 2019). We found a moderate correlation between plasma NFL levels and the MMSE score of patients, which supports its association with neurodegeneration and hence cognitive decline. However, its rise has also been reported in other neuronal injuries apart of AD (Meeter et al., 2016, Kovacs et al., 2017, Steinacker et al., 2018). In light of our own findings, we suggest the use of measuring blood levels of NFL to rule in or rule out neurodegeneration, in conjunction with P-tau for a more accurate diagnosis of AD.

### 4.1 Strengths and limitations

To the best of our knowledge, this is the first biomarker study on Alzheimer’s disease being reported from Pakistan. This study could become a ground breaker in the arena of AD research and pave the path for future disease modifying drug trials in our population. Once validated, plasma measurements of P-tau and NFL through ELISA can become practically useful and sustainable tests in the setting of a lower-middle country, unlike the use of ultrasensitive assays, which are scarcely available and are costly.

The selection of subjects for the study was based on the diagnosis of the attending neurophysician. Further, the differences in clinical assessment among different neurologists might have introduced selection bias. According to the NIA-AA, CSF markers should be used to confirm the diagnosis of Alzheimer's disease (Jack et al., 2018). However, in Karachi, we do not have any daycares for AD patients. These patients visit OPDs of healthcare facilities where lumbar punctures cannot be performed. Education level is reported to affect the MMSE scores of subjects. We used the already validated Urdu version of MMSE to overcome this limitation (Awan et al., 2015).

## 5 Conclusion

Our results support the use of plasma NFL and P-tau as biomarkers for AD. These biomarkers could be used to identify individuals at high risk of developing AD early in the course of the disease and serve as a pre-screener for referral to memory clinics. By enabling more precise diagnoses, these biomarkers could enhance therapeutic trials and improve overall treatment outcomes.

## Declaration of Competing Interest

The authors declare that they have no known competing financial interests or personal relationships that could have appeared to influence the work reported in this paper.
